# Hospital-level characteristics of the standardised mortality ratio for ischemic heart disease: a retrospective observational study using Japanese administrative claim data from 2012 to 2019

**DOI:** 10.7717/peerj.13424

**Published:** 2022-05-18

**Authors:** Ryo Onishi, Yosuke Hatakeyama, Kunichika Matsumoto, Kanako Seto, Koki Hirata, Yinghui Wu, Tomonori Hasegawa

**Affiliations:** 1 Department of Social Medicine, Toho University, Ota-ku, Tokyo, Japan; 2 School of Nursing, Shanghai Jiao Tong University, Shanghai, China

**Keywords:** Administrative claim data, Ischemic heart disease, In-hospital mortality, Quality indicator, Japan

## Abstract

**Background:**

Ischemic heart disease (IHD) is one of the leading causes of mortality worldwide and imposes a heavy burden on patients. Previous studies have indicated that the optimal care for IHD during hospitalisation may reduce the risk of in-hospital mortality. The standardised mortality ratio (SMR) is an indicator for assessing the risk-adjusted in-hospital mortality ratio based on case-mix. This indicator can crucially identify hospitals that can be changed to improve patient safety and the quality of care. This study aimed to determine the hospital-level characteristics of the SMR for IHD in Japan.

**Methods:**

This study was designed as a retrospective observational study using the Japanese administrative claim data from 2012 to 2019. The data of all hospital admissions with a primary diagnosis of IHD (ICD-10, I20-I25) were used. Patients with complete variables data were included in this study. Hospitals with less than 200 IHD inpatients in each 2-year period were excluded. The SMR was defined as the ratio of the observed number of in-hospital deaths to the expected number of in-hospital deaths multiplied by 100.The observed number of in-hospital deaths was the sum of the actual number of in-hospital deaths at that hospital, and the expected number of in-hospital deaths was the sum of the probabilities of in-hospital deaths. Ratios of in-hospital mortality was risk-adjusted using multivariable logistic regression analyses. The c-statistic and Hosmer-Lemeshow test were used to evaluate the predictive accuracy of the logistic models. Changes in SMRs in each consecutive period were assessed using Spearman’s correlation coefficient.

**Results:**

A total of 64,831 were admitted patients with IHD in 27 hospitals as complete submission data. The SMRs showed wide variation among hospitals, ranging from 35.4 to 197.6, and analysis models indicated good predictive ability with a c-statistic of 0.93 (95% CI [0.92–0.94]) and Hosmer-Lemeshow test of 0.30. The results of chi-square tests and *t*-tests for all variables to assess the association with in-hospital mortality were *P* < 0.001. In the analysis of trends in each consecutive period, the SMRs showed positive correlations.

**Conclusions:**

This study denoted that the SMRs for IHD could be calculated using Japanese administrative claim data. The SMR for IHD might contribute to the development of more appropriate benchmarking systems for hospitals to improve quality of care.

## Introduction

Ischemic heart disease (IHD) is one of the leading causes of mortality worldwide. In recent years, IHD was identified as the top of global causes of death and disability-adjusted life-years, especially in people aged 50 years and older. An overall trend for reduction in ischemic heart disease-related mortality has been observed; however, it remains an important disease in the aging society (GBD-NHLBI-JACC Global Burden of Cardiovascular Diseases Writing Group, 2020; Nowbar et al., 2019; GBD 2019 Diseases and Injuries Collaborators, 2020). In Japan, 70,082 people have died from IHD, especially acute myocardial infarction, which is a disease with a high mortality rate that imposes a heavy burden on patients (eStat, 2020; Finegold, Asaria & Francis, 2013; Okui, 2020; Khan et al., 2020).

In the past few decades, countless efforts have been made to improve the quality of acute care. Previous studies have indicated that the optimal care for IHD during hospitalisation may reduce the risk of in-hospital mortality and subsequent readmission (Nimptsch & Mansky, 2013; Centers for Medicine & Medicaid Services , 2020a; Centers for Medicine & Medicaid Services , 2020b). The hospital standardised mortality ratio is well-known as an indicator for assessing the risk-adjusted in-hospital mortality ratio based on case-mix (Canadian Institute for Health Information, 2019). This indicator can crucially identify hospitals that can be changed to improve patient safety and the quality of care. The risk-adjusted quality indicators in hospitals have been used in many developed countries and regions, such as the USA, the UK, Canada, Sweden, Australia, France, Singapore, and Hong Kong (Jarman et al., 2010; Scott et al., 2011; Aylin, Bottle & Majeed, 2007; Lingsma et al., 2018). For individual diagnostic groups, we could calculate the standardized mortality ratio (SMR) in hospital-level (Amin et al., 2019; Amin et al., 2020).

In Japan, hospital administrative data have been used broadly for research and quality improvement efforts. The Diagnostic Procedure Combination (DPC)/Per-Diem Payment System (PDPS) is a reimbursement system for acute care hospitals introduced in 2003, and the DPC database is an administrative claim and discharge abstract database for inpatient acute care. In the DPC/PDPS, Per-diem medical service reimbursement points for each DPC gradually decrease, in three stages. This is both because medical resources are used disproportionately during the early stage of a hospital stay and in order to stimulate earlier discharge and streamline medical care (Matsuda et al., 2011; Kitazawa et al., 2014; Hayashida et al., 2021). Administrative data, such as the DPC data, have been successfully used in designing health policies and disease management, as well as in analyzing healthcare processes and patient outcomes (Miyata et al., 2010; Shinjo & Fushimi, 2017; Amin et al., 2019; Amin et al., 2020).

This study aimed to develop a calculation methodology of SMRs for IHD using DPC data and clarify characteristics such as their trends in Japan. In designing the SMR calculation methodology, we considered that each hospital could easily evaluate it as an evaluation tool. To the best of our knowledge, this was the first large-scale study to calculate SMRs for IHD in Japan, revealing an 8-year trend using administrative data.

## Methods

This study was designed as a retrospective observational study using the DPC database. DPC data from the Medi-Target benchmarking project managed by the All Japan Hospital Association (AJHA) were used, in which patients and hospitals were anonymized, and authors didn’t know them. The AJHA is one of the largest nationwide hospital associations in Japan, comprising approximately 2,500 hospitals, which manages the Medi-Target project, a benchmark project using clinical indicators based on DPC data. Participation in the Medi-Target project was optional, 78 hospitals participated in 2020 (MEDI-TARGET, 2020).

All hospital admissions with a primary diagnosis of IHD from 2012 to 2019 were identified in the DPC database. The 10th revision of the International Statistical Classification of Diseases and Related Health Problems codes (ICD-10, I20-I25) were used to determine the primary diagnoses. Patients with complete variables data were included in this study. Hospitals with less than 200 IHD inpatients in each 2-year period were excluded. In this study, the main outcome was in-hospital death and it was defined death in whole hospitalization period based on previous studies (Canadian Institute for Health Information, 2019; Amin et al., 2019; Amin et al., 2020). In the SMR analyses, patient data included data on age, sex, admission urgency status (emergency or planned), use of ambulance, Charlson Comorbidity Index (CCI), and severity, which were used in previous studies (Centers for Medicine & Medicaid Services , 2020a; Centers for Medicine & Medicaid Services , 2020b; Amin et al., 2019; Amin et al., 2020; National Hospital Organization, 2020; Crowe et al., 2020). Age was considered a continuous variable, and sex, admission urgency status, use of ambulance, CCI, and severity were considered categorical variables. Since the crude patient mortality rates differed between hospitals of different function and capacity, requiring risk adjustment for patient severity (Sharma et al., 2021). Disease severity is commonly measured via the CCI or case-mix. In this study, additional measure was the patient severity level. In the DPC/PDPS, the patient clinical complexity level evaluates not only complications and comorbidities but also severity indices each corresponding disease.

The CCI was derived from secondary ICD-10 diagnosis codes. The CCI was a weighted score based on the number and type of diagnoses reported in the hospital summary data (Charlson et al., 1987; Sundararajan et al., 2004; Pylväläinen et al., 2019). In the DPC/PDPS, as for comorbidities, the top four serious comorbidities were reported. In this study, the CCI was calculated based on Quan’s modification (Quan et al., 2011) and was classified into three categories, CCI score 0 to 2, CCI score 3 to 4, and CCI score 5 or over (5+). The severity of IHD was classified using three indices: (i) New York Heart Association functional classification (NYHA) (Dolgin, 1994), (ii) Canadian Cardiovascular Society functional classification (CCS) (Campeau, 2002), and (iii) Killip’s classification (Killip) (Killip & Kimball, 1967). The NYHA score provided a simple way of classifying the extent of heart failure. It classifies patients into one of four categories (range 1–4: no limitation (1), slight limitation (2), marked limitation (3), severely limitation (4)) based on their limitations during physical activity. The CCS score grading of angina pectoris, a classification system used to grade the severity of exertional angina, classifies the severity of angina patients into one of four categories (range 1–4: no limitation (1), slight limitation (2), marked limitation (3), severely limitation (4)) based on subjective symptoms. The Killip score classifies acute myocardial infarction into four categories (range 1–4: no limitation (1), slight limitation (2), marked limitation (3), severely limitation (4)) based on the findings of pulmonary congestion and cardiogenic shock. It is known to be associated with the mortality rate after the onset of acute myocardial infarction. We defined severe patient as patient having a score of three or higher in at least one index (NYHA ≥ 3, CCS ≥ 3, or Killip ≥ 3). The IHD includes multiple diseases such as myocardial infarction and heart failure, and there was no single optimal index for assessing severity in DPC database. We collected data on patient’s condition at admission: NYHA, CCS, and Killip, all of which have clear restrictions on life in class 3 and over. For these reasons, we adopted a composite index and independently defined class 3 and over as severe. Outcome variable was the overall in-hospital mortality and referred to death that occurred at any point during the entire admission period based on previous studies (Canadian Institute for Health Information, 2019; Amin et al., 2019; Amin et al., 2020).

This study was based on a secondary analysis of administrative claims data. Owing to the anonymous nature of the data, no Institutional Review Board (IRB) approval was needed for this kind of study in Japan (Japanese Ministry of Education, Culture, Sports, Science and Technology, Japanese Ministry of Health, Labour and Welfare, 2019). This study was determined not to be applicable to ethical review by the Ethics Committee of the Toho University School of Medicine (No. A19053).

### Calculation methodology of SMRs

The SMR was defined as the ratio of the observed number of in-hospital deaths to the expected number of in-hospital deaths multiplied by 100. The observed number of in-hospital deaths was the sum of the actual number of in-hospital deaths at that hospital, and the expected number of in-hospital deaths was the sum of the probabilities of in-hospital deaths. An SMR above or below 100 indicates that the mortality rate is higher or lower, respectively, than the overall average.

A multivariable logistic regression analysis was constructed to predict the chance of in-hospital death for each patient with patient-level factors. Logistic regression analyses were performed to calculate the intercept of the covariates. The covariates for case-mix adjustments were age, sex, admission urgency status, use of ambulance, CCI, and severity. Coefficients derived from the logistic regression analyses were used to calculate the probability of in-hospital deaths. The sum of the predicted probabilities of in-hospital deaths (range, 0–1) provided the total expected number of in-hospital deaths in that hospital. The ratio of the observed number of in-hospital deaths and the expected number of deaths provided the standardised ratio for that hospital of interest.

\begin{eqnarray*}\mathrm{SMR}= \left( \frac{\text{Observed number of deaths in each hospital}}{\text{Expected number of deaths in each hospital}} \right) \times 100. \end{eqnarray*}

We constructed two SMR models. The first model for capturing trend of SMR used hospital data with complete 8-year submission. The second models for capturing SMR correlation between each consecutive period were using hospital data with each of 2-year period (2012–2013, 2014–2015, 2016–2017, and 2018–2019).

### Statistical analysis

The following variables were examined for their association with death: age, sex, admission urgency status, use of ambulance, CCI, and severity. Regarding the coefficient values, age was expressed in years, and sex, admission urgency status, use of ambulance, CCI, and severity were included as categorical variables. Continuous variables were summarized using descriptive statistics (mean ± standard deviation [SD]), whereas categorical variables were summarised as frequencies and proportions. Patient characteristics of dead or discharged groups were compared using chi-square tests for categorical variables and *t*-tests for continuous variable.

The c-statistic and Hosmer-Lemeshow test were used to evaluate the predictive accuracy of the logistic models. The c-statistic is derived by calculating the proportion of concordant pairs and is equivalent to the area under the Lorenz curve. C-statistics value of 0.5 suggests that the model is no better than random chance in predicting death and a value 1.0 indicates perfect discrimination. The relationships between the SMRs for each period in the 2-year data analyses were evaluated using Spearman’s correlation coefficient. *P* value <0.05 was considered statistically significant and no correction for multiple testing was performed.

All statistical analyses were performed using the Statistical Package for the Social Sciences, version 27.0.0.

### Patient and public involvement

This research was designed without patient involvement.

## Results

### Characteristics of the study population

Between April 2012 and March 2020, a total of 64,831 patients in 27 hospitals admitted with a primary diagnosis of IHD were included in the 8-year analysis. Table 1 shows the demographic characteristics of patients in the 8-year data and in the 2-year data. In the 2-year analyses, 60, 49, 39, and 28 hospitals were included in 2012–2013, 2014–2015, 2016–2017, and 2018–2019, respectively. The results of chi-square tests and *t*-tests for all variables to assess the association with in-hospital mortality were *P* < 0.001.

**Table 1:** Demographic characteristics of patients.

Characteristic		Discharged	Dead	*P* values^*^
Number of patients	n	64,102	729	<0.001
Number of hospitals	n	27	27	<0.001
Age	mean ± SD	70.1 ± 11.1	79.5 ± 10.8	<0.001
Sex (male)	n (%)	46,307 (72.2)	429 (58.9)	<0.001
CCI				<0.001
CCI score 0–2	n (%)	61,457 (95.9)	649 (89.0)	
CCI score 3–4		2,471 (3.9)	67 (9.2)	
CCI score 5+		174 (0.3)	13 (1.8)	
Admission urgency status (emergency)	n (%)	17,243 (26.9)	690 (94.7)	<0.001
Use of ambulance (use)	n (%)	9,218 (14.4)	523 (71.7)	<0.001
Severity (severe)	n (%)	8,991 (14.0)	448 (61.5)	<0.001

**Notes.**

n number of patients CCI Charlson comorbidity index *P* values two-sided significance

* Patient characteristics of dead or discharged groups were compared using chi-square tests for categorical variable and *t*-tests for continuous variable (Age).

In the 8-year data, 1.1% of the patients died in-hospital. The mean age of the admitted patients was 70.2 years, and 72.1% of these patients were male. Emergency admitted patients comprised 27.7% of the overall population, and 15.0% of patients used ambulance at admission. The emergency admission rate (acute rate) for each hospital ranged from 9.9% to 41.7%, and no hospitals were admitting elective patients only. Diabetes without chronic complication was the most common comorbidity, observed in 27.9% of patients at admission. The other associated comorbidities at admission included congestive heart failure (22.2%), peptic ulcer disease (10.3%), and history of myocardial infarction (8.5%).

### Characteristic of the SMRs

Table 2 shows the coefficients and significance of the variables of SMRs. All variables expected for sex showed a significant relationship with the in-hospital mortality. In the 2-year data analyses, age, urgency of admission, use of ambulance, and severity were also related to in-hospital mortality. The CCI score did not show a consistent relationship with mortality. Risk factors for both models were aging, urgency of admissions, use of ambulance, and high severity of angina, heart failure, or acute myocardial infarction.

The SMRs varied widely across the hospitals included in this study. Table 3 shows mean and SD of the SMRs in each period in 2-year data analyses. The SMRs ranged from 24.0–368.6, 25.5–426.4, 19.9–267.9, and 26.7–348.3 in 2012–2013, 2014–2015, 2016–2017, and 2018–2019, respectively.

**Table 2:** Variables for the logistic regression analysis for hospital standardised mortality ratio.

	OR (95% CI)	*P* values
Age	1.07 (1.06–1.08)	<0.001
Sex (male)	1.07 (0.91–1.26)	0.429
CCI score 0–2 (reference)		
CCI score 3–4	1.46 (1.12–1.92)	0.006
CCI score 5+	3.18 (1.67–6.05)	<0.001
Admission urgency status (emergency)	15.38 (10.79–21.92)	<0.001
Use of ambulance (use)	2.61 (2.18–3.12)	<0.001
Severity (severe)	4.13 (3.53–4.84)	<0.001

**Notes.**

CCI Charlson comorbidity index OR Odds ratio *P* values two-sided significance

**Table 3:** Mean and SD of the SMRs for each period in 2-year data analyses.

Period	n	Mean	SD
2012–2013	60	123.99	69.24
2014–2015	49	125.77	70.72
2016–2017	39	119.01	62.07
2018–2019	28	121.97	76.47

**Notes.**

SMR Standardised mortality ratio n number of hospitals SD standard deviation

**Figure 1:**
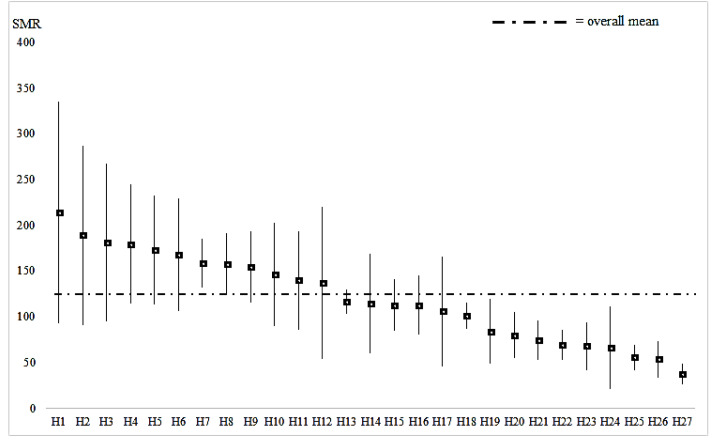
Mean and SD of the HSMR for each hospital (8-year data analysis, 27 hospitals).

C-statistics showed strong predictive ability for both models; *i.e.*, 0.93 (95% CI [0.92−0.94]) in the 8-year data analysis, and 0.93 (95%CI [0.92–0.95]), 0.93 (95%CI [0.91–0.94]), 0.93 (95%CI [0.92–0.95]), and 0.93 (95%CI [0.92–0.94]) in 2012–2013, 2014–2015, 2016–2017, and 2018–2019 in the 2-year data analyses, respectively. Moreover, the result of Hosmer-Lemeshow test revealed a significance of 8-year data analysis model; 0.30.

### Eight-year trend

The mean of SMR in the 8-year model was 120.2 suggesting that data of high SMR hospitals outweighed (Fig. 1); the percentage of hospitals with mortality higher than 100 SMR was 48.1% in 2012–2013, and 63.0% in 2018–2019. However, no consistent tendency was observed statistically for the SMR in the study period.

### Correlation of the SMRs in each consecutive period

In the 2-year data analyses, the correlation analyses revealed a significant positive relationship between the changes of SMRs in each consecutive period (Table 4). This indicated that high/low SMR hospitals were likely to continue over time.

## Discussion

This study indicated that SMRs for IHD can be calculated using DPC data in Japan. DPC/PDPS is a standard reimbursement system for acute care hospitals and almost all hospitals are submitting DPC data to the government, and the calculation method developed in this study could be used for assessing the quality of inpatient care for IHD. These results revealed that the SMRs for IHD varied considerably among hospitals with comparable case-mixes.

**Table 4:** Correlation between the SMRs in each consecutive period.

Period	n	r	*P* values
2012–2013–2014–2015	41	0.309	0.049
2014–2015–2016–2017	36	0.509	0.002
2016–2017–2018–2019	28	0.487	0.009

**Notes.**

SMR Standardized mortality ratio n number of hospitals r correlation coefficient (Spearman’s non-parametric correlation) *P* values two-sided significance

Positive correlation coefficient means that hospitals with lower/ higher HSMRs are likely to get same results in the following year.

In the 8-year data analysis, after the adjustments for case-mixes, some hospitals were found to have a higher mortality ratio. Regarding patient age, 72.6% of the admitted patients were 65 years and older. An older age contributed to an increase in the mortality ratio. It is well-known that aging contributes to the aggravation of IHD, and our results were consistent with previous studies (Nowbar et al., 2019). In this study, we observed no association between sex differences and in-hospital mortality; however, previous studies indicated that sex difference was a risk factor of IHD-related mortality studies (Nowbar et al., 2019). In the *t*-test results for relationship between mortality and sex were significant; however, there was not significant relationship between them in the logistic regression analysis adding other factors. It was suggested that in the influence on mortality, sex difference may be strongly influenced by other factors. The increased risk of mortality for admitted patients who were unplanned or transported by ambulance was similar to that found in previous studies (Sepehrvand et al., 2020), and the availability of critical care may affect the SMR.

In this study, we adopted the logistic regression analysis as risk adjusted method, because it was used by previous studies and easily to calculate in each hospital. Regarding risk-adjusting severity for IHD, we created a composite severity variable, which consisted of three related indices. The results obtained using this severity variable were useful for the calculation of SMR. The most remarkable finding of this study was that hospitals with high/low SMR were likely to produce the same results in the following period. This result suggested that the SMR for IHD was a stable indicator, and that there were underlying factors relating to SMR of the hospitals.

In the future studies, it is necessary to investigate the characteristics of good SMR hospitals so that poor SMR hospitals can get benefits in improving the quality of care. It is important for hospitals to be aware of the quality of their medical care, including management, and appropriate measures should be implemented. Benchmarking allows evaluation and comparison of quality of healthcare levels (Favez et al., 2020). The quality of medical care, especially reducing in-hospital mortality, is important for hospital management. In Japan, hospitals are obliged to publish some clinical indicators that may be expanded in the future. It also leads to the reputation of the hospital. Therefore, the SMR for IHD based on DPC data may be considered as an effective indicator for measuring the quality of medical care. As for Improving in-hospital mortality for poor SMR hospital, we thought that the hospital accreditation programs were effective. Therefore, it is conceivable to provide incentives for enrollment in hospital accreditation programs (Lee, Chun & Lee, 2021).

The strengths of this study included its large sample size, and relatively long 8-year study period. In addition, the risk-adjusted models were established and internally validated, and the results of c-statistics and Hosmer-Lemeshow test showed good predictive ability. However, this study was not free from limitations. First, participation to the Medi-target project was voluntary and might not represent the whole population in Japan. Additionally, large hospital had a large number of cases and might have stable SMR. These limitations may be the cause of a selection bias. Second, in the analyses, there was a limitation of using data. Since the clinical record in DPC data were voluntary reported and its sample size was very small, we were unable to use it. Other risk factors for mortality of IHD inpatients, such as BMI score, LDL- cholesterol level, and medical history, were not considered in this study. The hospital characteristics (*e.g.*, number of patients, location, and functions) which could not be examined because they were not included in the database used in this study might also have affected in-hospital mortality and acted as potential confounders. Furthermore, future studies should also consider newer statistical approaches such as random effects or machine learning models, which would result in more precise evaluations. Third, hospitals were changing their discharge policies to shorten the length of stay as a part of the health sector reform encouraged by the Ministry of Health, Labour and Welfare, Japan. Especially, the introduction of DPC/PDPS strengthened the incentives of early discharge, because DPC/PDPS regulated a standard length of hospital stay and there was no incentive for hospitals to provide long-term care (Wang et al., 2010; Hayashida et al., 2021). However, there was no significant change in the average length of hospital stay during this study (9.4 days, 2011; 8.4 days, 2014; 8.6 days, 2017). Forth, if patients were discharged to long-term care facilities or community services and died there, these instances were not counted as a death in the calculation of SMRs. In Japan, the integration of healthcare records and long-term care records is underway, and it is expected that comprehensive care analyses will be possible in the near future.

## Conclusions

The results of this study suggested that it was possible to use data from the DPC database to calculate the SMRs for IHD. The SMRs for IHD varied significantly among hospitals with comparable case-mixes. During the study period, the hospitals with high/low SMR were likely to produce the same results in the following period. The SMR for IHD using risk-adjusted and internally validated model might contribute to development more appropriate benchmarking systems for hospitals to improve their quality of care.

##  Supplemental Information

10.7717/peerj.13424/supp-1 Supplemental Information 1 Demographic characteristics of patients (2-year analyses)

10.7717/peerj.13424/supp-2 Supplemental Information 2 Variables for the logistic regression analysis for hospital standardised mortality ratio (2-year analyses)

## Abbreviations

IHD: ischemic heart disease
SMR: Standardised mortality ratio
DPC: Diagnostic Procedures Combination
PDPS: Per-Diem Payment System
NYHA: New York Heart Association functional classification
CCS: Cardiovascular Society functional classification
Killip: Killip’s classification
CCI: Charlson comorbidity index
ICD: International Classification of Diseases
IRB: Institutional Review Board
CI: confidence interval
OR: Odds ratio
SD: standard deviation

## Additional Information and Declarations

### Data Availability

The following information was supplied regarding data availability: The raw data cannot be shared publicly. External researchers can contact the Ethics Committee of Toho University regarding the use of data but the committee only accepts applications in Japanese (med.rinri@ext.toho-u.ac.jp, +81-3-3762-4151). If external researcher contacts the research team directly (health@med.toho-u.ac.jp, ryo.onishi@med.toho-u.ac.jp).

## References

[ref-1] Amin R, Hatakeyama Y, Kitazawa T, Matsumoto K, Fujita S, Seto K, Hasegawa T (2020). Capturing the trends in hospital standardized mortality ratios for pneumonia: a retrospective observational study in Japan (2010 to 2018). Environmental Health and Preventive Medicine.

[ref-2] Amin R, Kitazawa T, Hatakeyama Y, Matsumoto K, Fujita S, Seto K, Hasegawa T (2019). Trends in hospital standardized mortality ratios for Stroke in Japan between 2012 and 2016: a retrospective observational study. International Journal for Quality in Health Care.

[ref-3] Aylin P, Bottle A, Majeed A (2007). Use of administrative data or clinical databases as predictors of risk of death in hospital: comparison of models. BMJ.

[ref-4] Campeau L (2002). The Canadian Cardiovascular Society grading of angina pectoris revisited 30 years later.

[ref-5] Canadian Institute for Health Information (2019). Hospital Standardized Mortality Ratio (HSMR). https://www.cihi.ca/en/hospital-standardized-mortality-ratio-hsmr.

[ref-6] Centers for Medicine & Medicaid Services (2020a). Hospital 30-Day Acute Myocardial Infarction Readmission Measure methodology. https://www.cms.gov/Medicare/Quality-Initiatives-Patient-Assessment-Instruments/HospitalQualityInits/Measure-Methodology.

[ref-7] Centers for Medicine & Medicaid Services (2020b). Hospital-level 30-day all-cause unplanned readmission following coronary artery bypass graft surgery updated measure methodology report.

[ref-8] Charlson ME, Pompei P, Ales KL, MacKenzie CR (1987). A new method of classifying prognostic comorbidity in longitudinal studies: development and validation. Journal of Chronic Diseases.

[ref-9] Crowe F, Zemedikun DT, Okoth K, Adderley NJ, Rudge G, Sheldon M, Niranharakumar K, Marshall T (2020). Comorbidity phenotypes and risk of mortality in patients with ischemic heart disease in the UK. Heart.

[ref-10] Dolgin M (1994). Nomenclature and criteria for diagnosis of diseases of the heart and great vessels.

[ref-11] eStat (2020). Portal Site of Official Statistics of Japan. https://www.e-stat.go.jp/en/.

[ref-12] Favez L, Zuniga F, Sharma N, Blatter C, Simon M (2020). Assessing nursing homes quality indicators’ between-provider variability and reliability: a cross-sectional study using ICCs and rankability. International Journal of Environmental Research and Public Health.

[ref-13] Finegold JA, Asaria P, Francis DP (2013). Mortality from ischaemic heart disease by country, region, and age: Statistic from World Health organisation and United Nations. International Journal of Cardiology.

[ref-14] GBD 2019 Diseases and Injuries Collaborators (2020). Diseases and Injuries Collaborators. Global burden of 369 diseases and injuries in 204 countries and territories, 1990–2019: a systematic analysis for the Global Burden of Disease Study 2019. Lancet.

[ref-15] GBD-NHLBI-JACC Global Burden of Cardiovascular Diseases Writing Group (2020). Global burden of cardiovascular disease and risk factors, 1990-2019: update from the GBD 2019 study. Journal of the American College of Cardiology.

[ref-16] Hayashida K, Murakami G, Matsuda S, Fushimi K (2021). History and profile of diagnosis procedure combination (DPC): development of a real data collection system for acute inpatient care in Japan. Journal of Epidemiology.

[ref-17] Japanese Ministry of Education, Culture, Sports, Science and Technology, Japanese Ministry of Health, Labour and Welfare (2019). Ethical guidelines for epidemiological research. https://www.niph.go.jp/wadai/ekigakurinri/.

[ref-18] Jarman B, Pieter D, Veen AAvander, Kool RB, Aylin P, Bottle A, Westert GP, Jones S (2010). The hospital standardised mortality ratio: a powerful tool for Dutch hospitals to assess their quality of care?. BMJ Quality & Safety.

[ref-19] Khan MA, Hashim MJ, Mustafa H, Baniyas MY, Suwaidi SKBMAI, Alkatheeri R, Alblooshi FMK, Almatrooshi MEAH, Alzaabi MEH, Al Darmaki RS, Lootah SNAH (2020). Global epidemiology of ischemic heart disease: results from the global burden of disease study. Cureus.

[ref-20] Killip T, Kimball JT (1967). Treatment of myocardial infarction in a coronary care unit: a two year experience with 250 patients. The American Journal of Cardiology.

[ref-21] Kitazawa T, Matsumoto K, Fujita S, Yoshida A, Iida S, Nishizawa H, Hasegawa T (2014). Perioperative patient safety indicators and hospital surgical volumes. BMC Research Notes.

[ref-22] Lee BY, Chun YJ, Lee YH (2021). Comparison of major clinical outcomes between accredited and nonaccredited hospitals for inpatient care of acute myocardial infarction. International Journal of Environmental Research and Public Health.

[ref-23] Lingsma HA, Bottle A, Middleton S, Kievit J, Steyerberg EW, Marang-van de Mheen PJ (2018). Evaluation of hospital outcomes: the relation between length-of-stay, readmission, and mortality in large international administrative database. BMC Health Services Research.

[ref-24] Matsuda S, Fujimori K, Kuwabara K, Ishikawa KB, Fushimi K (2011). Diagnosis procedure combination as an infrastructure for the clinical study. Asian Pacific Journal of Disease Management..

[ref-25] MEDI-TARGET All Japan Hospital Association. https://www.ajha.or.jp/hms/dpc/.

[ref-26] Miyata H, Hashimoto H, Horiguchi H, Fushimi K, Matsuda S (2010). Assessment of hospital performance with a case-mix standardized mortality model using an existing administrative database in Japan. BMC Health Services Research.

[ref-27] National Hospital Organization (2020). Clinical evaluation index in the 2018 medical quality evaluation/publication promotion project. https://nho.hosp.go.jp/cnt1-1_00201.html.

[ref-28] Nimptsch U, Mansky T (2013). Quality measurement combined with peer review improved German in-hospital mortality ratios for four diseases. Health Aff.

[ref-29] Nowbar AN, Gitto M, Howard JP, Francis DP, Al-Lamee R (2019). Mortality from ischemic heart disease. Circulation: Cardiovascular and Outcomes.

[ref-30] Okui T (2020). Age-period-cohort Analysis of Cardiovascular Disease Mortality in Japan, 1995-2018. Journal of Preventive Medicine and Public Health.

[ref-31] Pylväläinen J, Talala K, Murtola T, Taari K, Raitanen J, Tammela TL, Auvinen A (2019). Charlson comorbidity index based on hospital episode statistics performs adequately in predicting mortality, but it’s discriminative ability diminishes over time. Clinical Epidemiology.

[ref-32] Quan H, Li B, Couris CM, Fushimi K, Graham P, Hider P, Januel JM, Sundararajan V (2011). Updating and validation the charlson comorbidity index and score for risk adjustment in hospital discharge abstract using data from 6 countries. American Journal of Epidemiology.

[ref-33] Scott IA, Brand CA, Phelps GE, Barker AL, Cameron PA (2011). Using hospital standardised mortality ratios to assess quality of care–proceed with extreme caution. Medical Journal of Australia.

[ref-34] Sepehrvand N, Alemayehu W, Kaul P, Pelletier R, Bello A, Welsh R, Armstrong P, Ezekowitz J (2020). Ambulance use distance and outcomes in patients with suspected cardiovascular disease: a registry-based geographic information system study. European Heart Journal. Acute Cardiovascular Care.

[ref-35] Sharma N, Schwendimann R, Endrich O, Ausserhofer D, Simon M (2021). Variation of daily care demand in Swiss general hospitals: longitudinal study on capacity utilization, patient turnover and clinical complexity levels. Journal of Medical Internet Research.

[ref-36] Shinjo D, Fushimi K (2017). The degree of severity and trends in hospital standardized mortality ratios in Japan between 2008 and 2012: a retrospective observational study. The International Society for Quality in Health Care.

[ref-37] Sundararajan V, Henderson T, Perry C, Muggivan A, Quan H, Ghali WA (2004). New ICD-10 version of the Charlson comorbidity index predicted in-hospital mortality. Journal of Clinical Epidemiology.

[ref-38] Wang K, Li P, Chen L, Kato K, Kobayashi M, Yamauchi K (2010). Impact of the Japanese diagnosis procedure combination-based payment system in Japan. Journal of Medical Systems.

