# Supplementary material for: Hospital-level characteristics of the standardised mortality ratio for ischemic heart disease: a retrospective observational study using Japanese administrative claim data from 2012 to 2019
Source: PeerJ. 2022 May 18;10:e13424. doi: 10.7717/peerj.13424 (PMC9123883; doi:10.7717/peerj.13424)
Supplement: Supplemental Information 1 [file peerj-10-13424-s001.docx]

**Table S1.** **Demographic characteristics of patients (2-year analyses)**

|  |  | 2012-2013 | | 2014-2015 | | 2016-2017 | | 2018-2019 | |
| --- | --- | --- | --- | --- | --- | --- | --- | --- | --- |
| Characteristic |  | Discharged | Dead | Discharged | Dead | Discharged | Dead | Discharged | Dead |
| Number of patients | n | 46,159 | 412 | 33,156 | 316 | 22,682 | 299 | 16,565 | 231 |
| Number of hospitals | n | 60 | 60 | 49 | 49 | 39 | 39 | 28 | 28 |
| Age | mean± SD | 69.4 ± 11.0 | 78.6 ± 11.2 | 69.9 ± 11.1 | 78.5 ± 10.6 | 70.2 ± 11.1 | 79.9 ± 11.4 | 70.6 ± 11.1 | 79.9 ± 11.0 |
| Sex (male) | n (%) | 33,129 (71.8) | 243 (59.0) | 23,774 (71.7) | 179 (56.6) | 16,386 (72.2) | 181 (60.5) | 11,949 (72.1) | 128 (55.4) |
| CCI |  |  |  |  |  |  |  |  |  |
| CCI score 0-2 | n (%) | 44,385 (96.2) | 364 (88.3) | 31,713 (95.6) | 276 (87.3) | 21.484 (94.7) | 254 (84.9) | 15,767 (95.2) | 213 (92.2) |
| CCI score 3-4 |  | 1,691 (3.7) | 43 (10.4) | 1,374 (4.1) | 33 (10.4) | 1,129 (5.0) | 33 (11.0) | 749 (4.5) | 15 (6.5) |
| CCI score 5+ |  | 83 (0.2) | 5 (1.2) | 69 (0.2) | 7 (2.2) | 69 (0.3) | 12 (4.0) | 49 (0.3) | 3 (1.3) |
| Admission urgency status (emergency) | n (%) | 12,001 (26.0) | 390 (94.7) | 8,732 (26.3) | 287 (90.8) | 5,982 (26.4) | 283 (94.6) | 4,657 (28.1) | 222 (96.1) |
| Use of ambulance (use) | n (%) | 5,586 (12.1) | 272 (66.0) | 4,413 (13.3) | 214 (67.7) | 3,079 (13.6) | 217 (72.6) | 2,536 (15.3) | 175 (75.8) |
| Severity (severe) | n (%) | 7,194 (15.6) | 283 (68.7) | 4,966 (15.0) | 217 (68.7) | 3,039 (13.4) | 192 (64.2) | 1,878 (11.3) | 131 (56.7) |

n = number of patients

CCI = Charlson comorbidity index
