# Supplementary material for: Hospital-level characteristics of the standardised mortality ratio for ischemic heart disease: a retrospective observational study using Japanese administrative claim data from 2012 to 2019
Source: PeerJ. 2022 May 18;10:e13424. doi: 10.7717/peerj.13424 (PMC9123883; doi:10.7717/peerj.13424)
Supplement: Supplemental Information 2 [file peerj-10-13424-s002.docx]

**Table S2.** **Variables for the logistic regression analysis for SMR (2-year analyses)**

|  | 2012-2013 | | 2014-2015 | | 2016-2017 | | 2018-2019 | |
| --- | --- | --- | --- | --- | --- | --- | --- | --- |
|  | OR (95% CI) | P values | OR (95% CI) | P values | OR (95% CI) | P values | OR (95% CI) | P values |
| Age | 1.07  (1.06-1.08) | <0.001 | 1.07  (1.05-1.08) | <0.001 | 1.07  (1.06-1.09) | <0.001 | 1.07  (1.05-1.08) | <0.001 |
| Sex (male) | 0.99  (0.80-1.23) | 0.953 | 0.88  (0.69-1.12) | 0.299 | 1.09  (0.84-1.42) | 0.502 | 0.90  (0.68-1.21) | 0.497 |
| CCI score 0-2 (reference) |  |  |  |  |  |  |  |  |
| CCI score 3-4 | 1.65  (1.17-2.32) | 0.004 | 1.54  (1.04-2.27) | 0.031 | 1.49  (0.99-2.24) | 0.056 | 1.02  (0.59-1.79) | 0.934 |
| CCI score 5+ | 4.00  (1.51-10.62) | 0.005 | 5.75  (2.34-14.13) | <0.001 | 9.08  (3.91-21.11) | <0.001 | 2.29  (0.61-8.55) | 0.219 |
| Admission urgency status | 16.31  (10.27-25.91) | <0.001 | 7.50  (4.83-11.65) | <0.001 | 14.42  (8.27-25.16) | <0.001 | 17.56  (8.52-36.19) | <0.001 |
| (emergency) |  |  |  |  |  |  |  |  |
| Use of ambulance  (use) | 2.50  (2.00-3.13) | <0.001 | 2.89  (2.20-3.80) | <0.001 | 2.81  (2.10-3.75) | <0.001 | 2.97  (2.13-4.15) | <0.001 |
| Severity (severe) | 4.69  (3.78-5.83) | <0.001 | 5.69  (4.43-7.31) | <0.001 | 4.60  (3.57-5.92) | <0.001 | 4.14  (3.14-5.46) | <0.001 |

SMR= Standardised mortality ratio

CCI= Charlson comorbidity index

OR= Odds ratio

P values= two-sided significance
